# Fragile X Premutation Associated Conditions (FXPAC)

**DOI:** 10.3389/fped.2020.00266

**Published:** 2020-05-27

**Authors:** Kirsten Johnson, Jonathan Herring, Jörg Richstein

**Affiliations:** ^1^The Fragile X Society, Great Dunmow, United Kingdom; ^2^Faculty of Law, University of Oxford, Oxford, United Kingdom; ^3^Interessengemeinschaft Fragiles-X e.V, Rostock, Germany

**Keywords:** Fragile X, premutation, FXTAS, FXPOI, FXPAC

## Abstract

The European Fragile X Network (EFXN) proposes that Fragile X Premutation Associated Conditions (FXPAC) be adopted as a universal term covering any condition linked to the Fragile X premutation. To date, there has not been an umbrella term assigned to issues associated with the FMR1 premutation, though several defined conditions which affect some premutation carriers, namely Fragile X-associated Primary Ovarian Insufficiency (FXPOI) and Fragile X-associated Tremor/Ataxia Syndrome (FXTAS), are now commonly accepted. An overarching term covering all FX premutation conditions will help doctors in determining how the premutation might be affecting their patient; and encourage researchers to explore the interrelationships of the various conditions affecting premutation carriers. Further, there are ongoing discoveries about physical and psychological issues faced by premutation carriers, and a new term helps encompass all of these burgeoning developments.

Fragile X Syndrome (FXS) is the most common form of inherited learning difficulty (1) and has been estimated to affect one in every 2,500–7,000 males and one in 2,500–11,000 females (2). Those with FXS typically have an expanded CGG repeat of over 200 in the 5′ untranslated region of FMR1 at Xq27.3. The general population have an average of 30 CGG repeats; those who have between 55 and 200 repeats are deemed to have the premutation (3). The precise presentation of the conditions varies. Most males with FXS have intellectual disability and behavior features including anxiety, attention deficit and symptoms of being on the autism spectrum. The symptoms for females with FXS range from mild to severe, and include learning difficulties, social skills and communication challenges, and mental health issues (4). For females who are less affected, whilst their difficulties are more subtle, they as a result face all the challenges associated with that of hidden disability.

Initial research into FXS assumed that the only significance for being a carrier was that their children might inherit the gene. However, it is now clear that the health issues for carriers go beyond the impact on the progeny.

Research is ongoing into the conditions which affect Fragile X premutation carriers. Established conditions associated with the premutation include Fragile X-associated Tremor/Ataxia Syndrome (FXTAS) (5) and Fragile X-associated Primary Ovarian Insufficiency (FXPOI) (6). FXTAS is a late-onset neurodegenerative condition with symptoms typically displaying after the age of fifty and worsening with age. It presents more commonly in men, with around 40% of male premutation carriers developing FXTAS and 16% of females. Characteristic symptoms include intention tremor, as well as difficulty with coordination and balance (ataxia), although not everyone with FXTAS evidences both features. Diagnosis is made based upon a combination of clinical and radiological examinations (7).

FXPOI is seen in around 20% of women with the *FMR1* premutation. Indeed, the Fragile X premutation is the most frequent single gene cause of primary ovarian insufficiency (8). FXPOI is characterized by reduced indicators of ovarian function (such as unpredictable or absent menses) and an impaired ovarian response. On average, the age of menopause is five years younger in women with the Fragile X premutation than the general population (9).

Research has shown people who are premutation carriers experience some mental and physical health symptoms at higher rates than in the general population (10). These are not encompassed by FXTAS or FXPOI. These additional impacts of being a carrier have been identified through studies with people who are aware that they are Fragile X premutation carriers (typically with a child with Fragile X Syndrome); and, more recently, in research which involved looking at the health records of a large sample of people in the general population, some of whom were premutation carriers (11). In 2018, the term Fragile X-associated Neuropsychiatric Disorders (FXAND) has been suggested to cover a range of neuropsychiatric and physical conditions associated with the Fragile X premutation. Neuropsychiatric conditions are said to affect approximately half of premutation carriers. Anxiety and depression are the most common issues seen in adults, though researchers have argued that physical problems such as chronic pain and autoimmune difficulties can heighten neuropsychiatric issues (12).

It is clear that we are only beginning to understand the wider significance of being a Fragile X premutation carrier and further research is needed to understand all the possible consequences. This is necessary to ensure there is effective recognition, understanding, and support for all people with the Fragile X premutation.

The European Fragile X Network (EFXN), at their meeting in Rotterdam, The Netherlands, on 29 September 2019, had a symposium on recent research on conditions which affect Fragile X premutation carriers. EFXN represents 17 family organizations across 16 countries in Europe, and consists of volunteers who advocate on behalf of those with Fragile X and associated conditions. Many of these volunteers carry the Fragile X premutation and are therefore directly affected by any terminology used.

There has been dismay over the past year at the suggested term Fragile X-associated Neuropsychiatric Disorder (FXAND). This term includes both physical and psychiatric symptoms under one umbrella (12). Many females carrying the premutation testified to the stigma they felt when labeled with having a “disorder.”

In their discussions, EFXN members identified several concerns:

- The term FXAND (Fragile X-associated Neuropsychiatric Disorder), by including auto-immune conditions and neuropsychiatric conditions under a single term, was not well-defined. It grouped together a wide variety of conditions. The term seemed to the EFXN representatives to be used as a “catch all” to refer to conditions that were not covered by FXTAS and FXPOI. While it might be helpful to have a term that referred to all conditions that might impact a carrier or a term that referred to a tightly defined set of conditions, FXAND as defined in the 2018 article was neither of these.
- The inclusion of the word disorder in the term FXAND was seen as stigmatizing of the premutation carrier. The conditions that fall under the current term FXAND are various, including social phobia, sensitivity to external stimuli, and depression. At the meeting, such characteristics were not seen as disorders but a common part of the human condition for many. The term disorder portrayed the impact of being a carrier in a necessarily poor light and could induce social or professional exclusion. Many premutation carriers are high-functioning with university degrees and established careers. They do not regard themselves as “disordered.” Using “condition” rather than “disorder” would be much preferred by the EFXN as it is broader language and keeps open a discussion about the desirability of the state.

After much debate, both on the day and then later in consultation with researchers, particularly those who were advisors to the Fragile X groups, it was decided to propose the umbrella term Fragile X Premutation Associated Conditions (FXPAC). This refers to all conditions affecting the premutation carrier. FXPAC does not replace FXTAS or FXPOI, rather FXTAS and FXPOI are specific examples of FXPAC. Further, FXAND should be replaced with two terms: Fragile X-associated Neuropsychiatric Conditions (FXANC); and Fragile X Various Associated Conditions (FXVAC), to cover other physical conditions that are not neuropsychiatric in nature. These suggestions are motivated by three main reasons.

First, the term FXPAC is a helpful overarching term to describe all conditions that might be associated with being a premutation carrier. We are still learning what all these conditions are and how they interrelate with each other and can interact. By having one label for them all researchers can be encouraged to explore their relationship further.

Second, the EFXN representatives found the term FXAND as being a confusing collection of physical and psychiatric conditions. The symptoms associated with FXPAC will include those linked to FXTAS and FXPOI. The neuropathology of FXTAS is well-established (13) and the clinical symptoms clearly differentiated (14, 15). FXPOI also has neat diagnostic indicators for clinical diagnosis (16). Recent research has looked at the clustering of symptoms in 355 females with the premutation (17). Though research is ongoing, the following symptoms (in addition to those associated with FXTAS or FXPOI) have been found to occur in higher rates in people with the Fragile X premutation:

- Anxiety, and anxiety disorders
- Low mood and depression
- Elevated traits related to autism, such as differences in the processing of social information and use of social (pragmatic) language. Of note, this refers to subtle characteristics that occur to varying degrees in the general population rather than symptoms of autism, *per se*. Although, in a small number of individuals a diagnosis of autism might be appropriate.
- Physical health symptoms such as chronic fatigue, chronic pain, fibromyalgia, autoimmune disorders, and sleep problems have also been identified at higher rates in people with the Fragile X premutation.

It is not helpful to have FXAND cover such a broad range of conditions. The proposed term FXANC would be narrower and be limited to neuropsychiatric conditions. FXVAC can be used for other physical conditions. The new terms FXANC and FXVAC would have more clearly defined boundaries and meanings than the “catch all” term of FXAND.

Third, the proposed terminology avoids the label “disorder.” The European premutation carriers felt strong that the word condition is a more accurate and less stigmatizing word to use for these conditions. We do recognize that in some countries the term disorder has benefits in strengthening arguments that treatment for it should be funded under health insurance. However, these financial considerations must be weighed against the stigma facing those with the condition. Further, it is hard to believe that the label used will impact on whether an insurance policy covers a particular condition or not.

EFXN, therefore, proposes Fragile X Premutation Associated Conditions (FXPAC) to be an overarching term to cover issues relating to being a carrier. Under that general heading, specific conditions will be:

- FXTAS (Fragile X-associated Tremor/Ataxia Syndrome)
- FXPOI (Fragile X-associated Primary Ovarian Insufficiency)
- FXANC (Fragile X-associated Neuropsychiatric Conditions), replacing the word “disorder” in FXAND, with “conditions”;
- FXVAC (Fragile X Various Associated Conditions) for any other non-psychiatric conditions (such as auto-immune conditions, chronic fatigue, fibromyalgia, etc.) which should not be included in FXANC but referred to as FXVAC (Figure 1).

**Figure 1:**
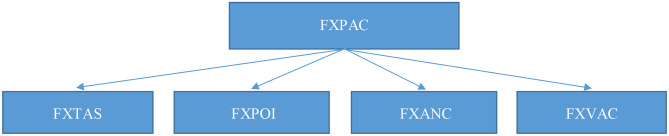
FXPAC, Fragile X Premutation Associated Conditions; FXTAS, Fragile X-associated Tremor/Ataxia Syndrome; FXPOI, Fragile X-associated Primary Ovarian Insufficiency; FXANC, Fragile X-associated Neuropsychiatric Conditions; FXVAC, Fragile X Various Associated Conditions.

Further research might define these terms more precisely and/or add to them. Also, the inter-relation between the various FXPAC conditions is not understood, with some carriers seemingly being unaffected, and others having a combination of conditions affecting them.

The European Fragile X Network hopes that researchers and clinicians will adopt FXPAC when generally referring to conditions that may affect premutation carriers. Having a unified term FXPAC gives a doctor, when helping someone with the premutation, a starting point for looking at the array of conditions which may or may not affect a particular premutation carrier. Further, it will encourage researchers to explore the interactions between these conditions.

It is important to highlight that many of the FXPAC symptoms are also common in the general population. However, the emerging research suggests that people with the premutation may be at higher risk of some of the symptoms. In addition, for many, these traits may present in subtle ways that do not affect day-to-day life. Though, a proportion of people who are Fragile X premutation carriers will experience symptoms to the extent it has a negative impact for them. Being a carrier does not mean that these symptoms are inevitable, and treatment should be sought for these symptoms as for anyone in the general population.

In conclusion, there is a need for a term which encompasses conditions affecting Fragile X premutation carriers. The EFXN, covering a population of over 500 million people throughout Europe, has worked together to come up with new terminology. FXPAC (Fragile X Premutation Associated Conditions) has been proposed to describe the wide range of symptoms which may affect premutation carriers. This has been welcomed by European researchers and clinicians.

FXPAC will aid researchers who wish to explore the many ways the premutation affects carriers, allowing for the fact we do not yet understand how these may be interlinked. FXPAC will help carriers who experience a range of conditions and wish to have a medical diagnosis. FXPAC will be a one-stop shop for doctors who need to explore the various conditions which may affect their patient. The network of European Fragile X family organizations very much hopes these ideas will be widely adopted outside Europe and around the world.

## Author's Note

Information on the work of the European Fragile X Network may be found at www.fragilex.eu. This is a group of charitable organizations who support families and those affected by Fragile X across Europe.

## Author Contributions

This article was drafted by KJ, revised by JH, with additional contributions from JR.

## Conflict of Interest

The authors declare that the research was conducted in the absence of any commercial or financial relationships that could be construed as a potential conflict of interest.
